# Streamlining event extraction with a simplified annotation framework

**DOI:** 10.3389/frai.2024.1361483

**Published:** 2024-04-29

**Authors:** Chanatip Saetia, Areeya Thonglong, Thanpitcha Amornchaiteera, Tawunrat Chalothorn, Supawat Taerungruang, Pakpoom Buabthong

**Affiliations:** ^1^Kasikorn Labs, Kasikorn Business-Technology Group, Nonthaburi, Thailand; ^2^Department of Thai, Faculty of Humanities, Chiangmai University, Chiang Mai, Thailand; ^3^Faculty of Science and Technology, Nakhon Ratchasima Rajabhat University, Nakhon Ratchasima, Thailand

**Keywords:** event extraction, annotation guideline, Universal Dependencies, generative model, event graph

## Abstract

Event extraction, grounded in semantic relationships, can serve as a simplified relation extraction. In this study, we propose an efficient open-domain event annotation framework tailored for subsequent information extraction, with a specific focus on its applicability to low-resource languages. The proposed event annotation method, which is based on event semantic elements, demonstrates substantial time-efficiency gains over traditional Universal Dependencies (UD) tagging. We show how language-specific pretraining outperforms multilingual counterparts in entity and relation extraction tasks and emphasize the importance of task- and language-specific fine-tuning for optimal model performance. Furthermore, we demonstrate the improvement of model performance upon integrating UD information during pre-training, achieving the F1 score of 71.16 and 60.43% for entity and relation extraction respectively. In addition, we showcase the usage of our extracted event graph for improving node classification in a retail banking domain. This work provides valuable guidance on improving information extraction and outlines a methodology for developing training datasets, particularly for low-resource languages.

## 1 Introduction

The advent of large language models (LLMs) has enabled significant progress in the field of natural language processing (NLP) and has helped provide promising results for various tasks (Brown et al., 2020). Many types of LLM have been proposed to solve both language-specific and domain-specific tasks (Lewis et al., 2019; Chung et al., 2022; Touvron et al., 2023). However, LLMs primarily favored well-resourced language with large updated training corpora, which may lead to hallucination problems, especially in lower-resource languages, in which the training corpora are not abundantly available (Ji et al., 2023). Extracting knowledge from these low-resource languages is not only beneficial as it helps include more available data. It could also provide deeper insight into the model's behavior across linguistic variations. To mitigate the hallucination problem, researchers have explored augmenting LLMs with external structured data sources, such as knowledge graphs (Guu et al., 2020; Asai et al., 2021; Mialon et al., 2023). Integrating structured information graphs with the LMs has been one of the common approaches (Yao et al., 2019; Kang et al., 2022), as graphs can be constructed in a domain-specific fashion, such as finance (Yang et al., 2018; Elhammadi et al., 2020).

Event graphs, which store event information from unstructured plain texts that describe “who, when, where, what, why” and “how” of the action, can provide a simplified version of a more generalized knowledge graph (Xiang and Wang, 2019; Li et al., 2022). Focusing on event extraction is particularly promising for enhancing NLP in low-resource settings because it involves parsing relationships within the narrow scope of particular events, thus requiring less extensive linguistic understanding for the model. Although close-domain event extraction, which follows specific domain schema, may provide better results in downstream retrieval tasks (Chambers et al., 2014; Björne and Salakoski, 2018; Han et al., 2018), this specialization often results in complex annotation systems that can be cumbersome and domain-restrictive, especially for low-resource languages. Moreover, while the use of additional syntactic information for extraction tasks has been studied in English (Fader et al., 2011; Wang C. et al., 2023; Wang Z. et al., 2023), it remains under-explored in low-resource languages.

In this work, we propose a methodology that streamlines the process for open-domain event extraction for corporate documents written in Thai and demonstrates its utility in a downstream task. Our guideline aims to make structured information extraction more accessible, by reducing the complexity of the annotation process. We also utilize Universal Dependencies [UD; Nivre et al., 2016] during the pre-training step to help the extraction model better understand the structural information of the sentences.

The main contributions of this work are as follows:

- **Annotation framework:** We offer a simplified annotation guideline that streamlines the event extraction process and presents a comparative analysis with the traditional Universal Dependency (UD) framework.
- **Event extraction models:** We explore the impact of language-specific and task-specific pre-training as well as the incorporation of UD on the improvement of the overall extraction performance.
- **Applications:** We demonstrate that the extracted event graph can be utilized to improve a downstream task, namely, node classification in a retail banking domain.

The rest of this paper is organized as follows. Section 2 analyzes previous work. Section 3 describes our methodology. Section 4 reports on our experiments. Section 5 provides a discussion of the results. Section 6 elaborates on the application of the event graphs. Section 7 concludes with a summary. By simplifying the initial extraction process, our method could allow for a more straightforward transition into an extraction task for other types of relations, such as, part-of or causal relations, which often require a deeper understanding of the interconnectedness of entities beyond their basic semantic relationships.

## 2 Related work

In this section, the background of the paper is explained along with literature reviews, outlining the previous work on event extraction, and Universal Dependencies. First, the definition and prior works of event extraction are explained. Second, the Universal Dependencies are described including the definition and its advantages.

### 2.1 Event extraction

Event extraction typically aims to extract event attributes from a raw, answering the 5W1H (who, what, when, where, why, and how) questions (Xiang and Wang, 2019). In earlier work, event extraction is considered a sequence labeling-based task (Gupta and Manning, 2014; Chen et al., 2020). The event trigger and its arguments are extracted as a span of words with an inside-outside-beginning (BIO) tagging system (Li et al., 2022). However, multiple events may be found in a given sentence, thus later necessitating the classification of the relation between each argument with its trigger.

The event extraction task can generally be categorized into two groups: close domain and open domain (Xiang and Wang, 2019; Liu et al., 2021, 2023). The close-domain extraction aims to extract a pre-defined structure based on supervised datasets. Most approaches first identify the event trigger, followed by its corresponding attributes (Huang et al., 2017; Xiang and Wang, 2019). Each event attributed is connected to the trigger with a pre-defined relation.

Various methods were proposed to address close-domain extraction (Chen et al., 2015a; Huang et al., 2017; Li et al., 2020). Some treat the event extraction as a sequence of sub-tasks: trigger identification, Trigger classification, argument identification, and argument role classification (Chen et al., 2015b; Yang et al., 2019; Li et al., 2022). However, this technique could lead to error propagation during the process (Li et al., 2019; Zhang et al., 2019). To minimize this error propagation, joint-trained models were proposed (Hsu et al., 2021; Lu et al., 2021). Many approaches adopt deep learning model architecture to train an end-to-end event extraction (Nguyen and Nguyen, 2019; Wadden et al., 2019). Recently, conditional generations from language models yield promising accuracy among many NLP tasks. Such models have been adopted for event extraction, achieving state-of-the-art accuracy over the complex classification models (Hsu et al., 2021; Lu et al., 2021). Nevertheless, the learning for these deep learning model approaches is supervised, necessitating a large amount of training data, which is not practical for low-resource languages.

Although the accuracy of the close-domain models is promising, most datasets are still limited to specific domains like medical data, historical documents, or specific types of news (Vanegas et al., 2015; Björne and Salakoski, 2018; Han et al., 2018). Thus, to extract a generic event from more generalized corpora, open-domain event extraction was developed (Chau et al., 2019; Liu et al., 2019). The early model considers the headline phrase as an event and disambiguates the events using Wordnet (Miller, 1995) and word sense disambiguation (Chau et al., 2019). This method leads to suboptimal performance as the arguments of an event are not necessarily positioned next to an event trigger keyword. To address this limitation, another model utilizes an unsupervised method using a neural latent variable model to extract an event (Liu et al., 2019). However, because of its unsupervised architecture, this method is not controllable and can extract an inaccurate event entity.

### 2.2 Low-resource event extraction

Similar to other tasks under a low-resource setting, the development of event extraction for low-resource languages generally focuses on methods that require less amount of training data. Zero-shot learning is one of the most common approaches to help the model perform tasks without additional training samples. Previous work on zero-shot event extraction has explored the use of representation in other latent spaces such as semi-Markov conditional random fields (Lu and Roth, 2012), Abstract Meaning Representation (AMR; Huang et al., 2018), pre-defined ontological structure (Zhang et al., 2021). Alternatively, event extraction tasks may be formulated as different tasks such as question-answering (Lyu et al., 2021). However, these techniques necessitate that proficient models already exist in the target language.

On the other hand, few-shot learning can be utilized to minimize the amount of new training data that is specific to the extraction tasks, while improving the overall performance of the models. Early models use a prototypical network to classify the extracted token (Snell et al., 2017; Lai and Nguyen, 2019), or minimize the supervised training data by providing the trigger terms in the annotation guideline as seeds for each event type (Bronstein et al., 2015). More recent work addresses the issue of low sample diversity by introducing Adaptive Knowledge-Enhanced Bayesian Meta Learning (AKE-BML) that uses a prior knowledge distribution to generate the posterior distribution for each event type (Shen et al., 2021). Techniques used in a few-shot setting typically work well when there exists a known distribution within a given task followed by model refinement through additional examples in the target tasks. For example, in Thai, we can pre-train the model with a syntactic structure such as UD, then fine-tune the model with a small number of labels for event extraction.

Furthermore, cross-lingual transfer may be employed when both languages have well-established parallel corpus. Recent methods have proposed transferring the entire universal structures across languages (Li et al., 2016; Subburathinam et al., 2019; Lou et al., 2022), or leveraging multilingual embedding when training the extraction model (M'hamdi et al., 2019). However, the cross-lingual approach typically requires extensive lexical mapping which may not be suitable for this initial stage of the development.

### 2.3 Relation extraction

In addition to models specific to event extraction, other relation extraction models may be utilized. End-to-end deep learning models have been proposed to concurrently extract entities and relation (Bekoulis et al., 2018; Eberts and Ulges, 2019; Hang et al., 2021). SpERT (Eberts and Ulges, 2019), in particular, has shown promising results on both entity and relation extraction evaluated over the SciERC dataset (Luan et al., 2018).

Moreover, generative pre-trained language models have been reported to achieve high performance on many NLP tasks (Brown et al., 2020; Touvron et al., 2023). Structured prediction using generative LMs, in particular, has recently attracted interest, due to their flexibility and applicability to new datasets. Most models are trained to generate structured output for named entities recognition or relation extraction from unstructured texts (Eberts and Ulges, 2019; Lu et al., 2021; Paolini et al., 2021). DeepStruct (Wang C. et al., 2023), for example, offers state-of-the-art performance when predicting the triplet from various domains, namely T-REx (Elsahar et al., 2018), TEKGEN, KELM (Agarwal et al., 2021), WebNLG (Colin et al., 2016), and ConceptNet (Speer et al., 2017).

Nevertheless, the models may not perform well in other languages that are not primarily present in the pre-training dataset. Other syntactic or semantic information, such as Universal Dependencies (UD) may assist in cross-lingual transfer of the extraction capabilities.

### 2.4 Universal Dependencies

Universal Dependencies (UD; Nivre et al., 2016) is a cross-language framework that allows for consistency in the annotation of syntactic grammatical structure (parts of speech, morphological features, and syntactic dependencies). Given this UD, a reliable graph can be created to represent the syntactic structure of an arbitrary text. Some event extraction models have been reported to benefit from the incorporation of UD (Björne and Salakoski, 2018; Chau et al., 2019). Unsupervised techniques can extract phrases and their relation from the UD graphs (Chau et al., 2019). Other work used the output of the UD as a graph feature along with a graph neural network to improve an event extraction model (Liu et al., 2018; Ahmad et al., 2021). Nevertheless, developing extraction models that rely too heavily on UD may pose similar limitations to those with languages that have low annotated training data, since the models may learn to capture only the explicit syntactic relationship and not the generalized semantic structure of the sentences.

## 3 Methodology

This section outlines the annotation process and the event extraction models used in this work.

### 3.1 Annotation framework

Frameworks for annotating text typically have two distinct aspects: (1) the practical means of how to annotate, and (2) the rules governing the annotation process (Pyysalo et al., 2012; Stenetorp et al., 2012; Cassidy et al., 2014). For (1), in this work, we configured INCEpTION (Klie et al., 2018) for entity and relation tagging. For (2), the complete annotation guideline is provided in the Supplementary material, while the abbreviated version, along with the design reasoning, is presented as follows.

Briefly, instead of the traditional event annotation where the trigger verb is identified first, the events are tagged based on 5W1H questions. The annotation guideline proposed two-stage tagging, which first labels entity spans and then links the relations among them. An example of a fully annotated sentence is shown below.


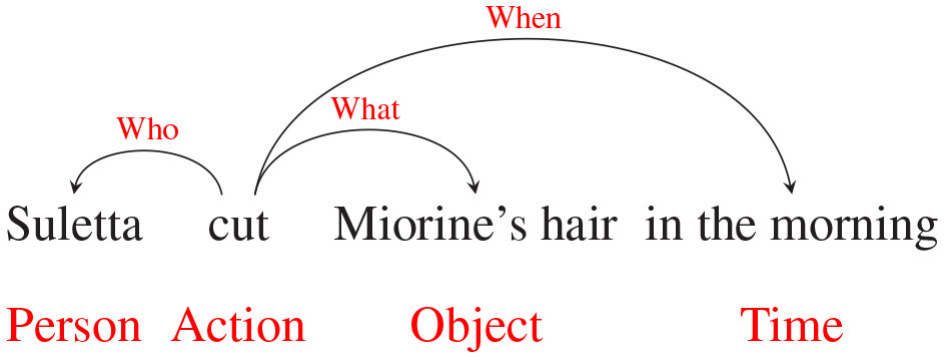


Entities, which are graph nodes of an event graph, are extracted as triggers and their corresponding arguments, represented as word spans. These entity spans are categorized into seven types to include the semantic meaning of an entity. One of the types is denoted as *Action*, which is similar to the trigger of an event. Other types are the semantic type of the argument, like *Person, Object*, and *Location*.

After getting entity spans, the subsequent step is establishing and classifying the relations among the spans. The classified relation types are designed to primarily address WH questions, which are *what, who, when*, and *where*. The *how* and *why* are not included, since the phrase that describes these two relations can be highly subjective depending on the annotator. Nevertheless, we also include additional relations, namely, same-unit, benefit, and value, in the guideline as these relations are not semantically ambiguous and can be potentially useful for downstream information extraction tasks.

### 3.2 Event extraction models

Two candidate models are selected for the event extraction task based on their inference settings: generative and span-based classification.

Span-based joint entity and relation extraction. The two models, SpERT (Eberts and Ulges, 2019) for span-based classification and DeepStruct (Wang C. et al., 2023) for the generative approach, were selected based on their demonstrated state-of-the-art performance in their respective tasks. SpERT has shows superior performance in span-based classification tasks, benchmarked on CoNLL-2003 (Tjong Kim Sang and De Meulder, 2003). Similarly Deepstruct has exhibited strong performance using generative approach on ACE-2005 (Walker and Consortium, 2005) corpus due to their superior performance in their respective tasks.

#### 3.2.1 Span-based classification model

For the baseline model, SpERT is used to represent a relatively more straightforward approach to the event extract task. In this approach, the model first recognizes the spans of the token of interest (entity extraction), then, with each pair of spans, learns to classify the relation types (relation extraction). Nevertheless, both entity extraction and relation extraction are trained jointly.

#### 3.2.2 Generative model

**Figure 1:**
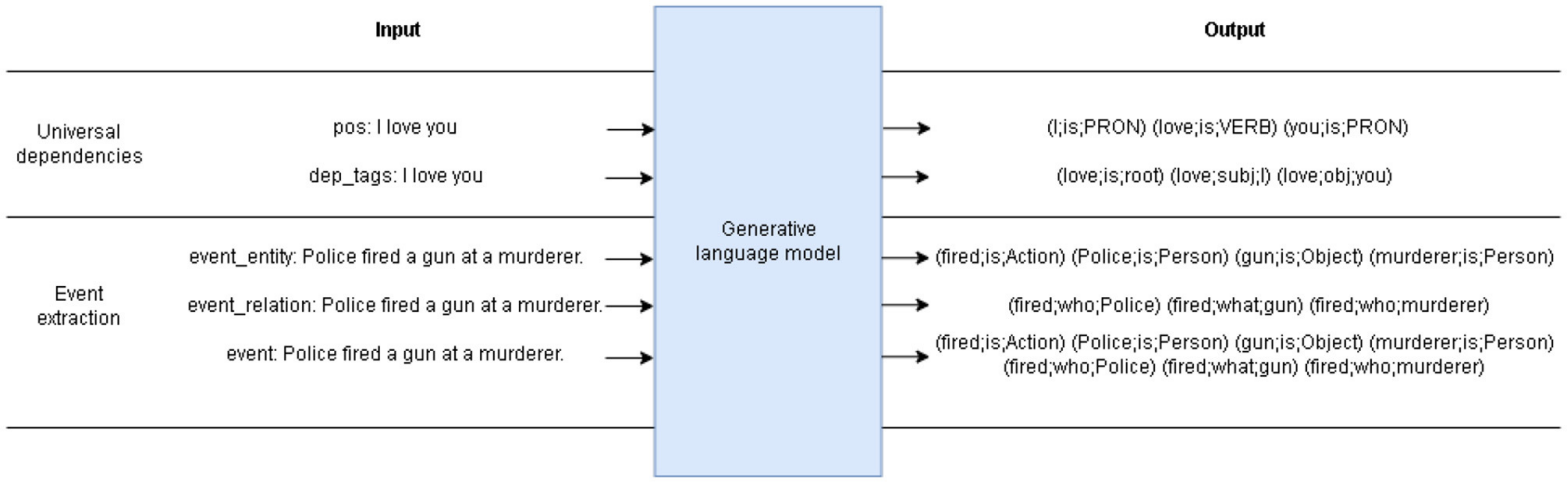
An schematic showing input and output of each generative task.

To study the effect of incorporating UD structure into the model, a separate model based on DeepStruct is used. The model is trained in a generative setting using a short prompt and the text of interest as the input, with the event triplets as the output (shown in Figure 1) In the UD pre-training, two tasks are trained jointly but with different prompts: part-of-speech (POS) tagging, and dependency (DEP) tagging. In contrast to the previous work where the extracted triplets are only constrained to a few important relations, each word in the input sentence of our approach will result in its own POS and DEP triplets. Note that in this generative setting, both entity extraction and relation extraction are inferred simultaneously from the model.

Since the original model is pre-trained only with the English dataset, herein we pre-trained the model on our Thai dataset (UD). After the pre-training process, the model is fine-tuned on the annotated Thai event dataset. Similar to the pre-training stage, the three tasks are trained jointly using different prompts and outputs.

## 4 Experiments and results

In this section, we compare the annotation time between event annotation using our proposed guideline and the traditional UD annotation. The annotated data was then used in a comparative study between different approaches to event extraction tasks.

### 4.1 Time for annotation

Our proposed guideline was used to annotate news articles and internal corporate documents written in Thai. To measure the time for annotation, two annotators were tasked to label the documents according to our guidelines as well as the standard Thai UD annotation for 1 month. Afterward, the number of annotated sentences for each task was divided to calculate the daily average from both annotators was averaged per day and divided by the number of days in that month. The statistics of the resulting annotated data are shown in Table 1.

**Table 1:** The data statistics of the annotated dataset of event extraction.

**Statistic**	**Train**	**Validation**	**Test**
# sentences	13,566	2,000	2,000
# entities	15,306	2,173	2,073
# relations	9,117	1,295	1,223

The number of sentences annotated using our event extraction guideline compared to using the typical UD guideline are 292.77 and 19.2 sentences per day, respectively, indicating ~10 times faster annotation speed.

### 4.2 Event extraction model

The annotated event dataset was used to evaluate event extraction models described in this section. First, the dataset is split into a train, validation, and test dataset with ratios of 0.78, 0.11, and 0.11, respectively (we allocated 2,000 sentences each to the validation and test split and used the remaining for the training). To evaluate the model, the micro-average F1 score, calculated separately between the entities F1 score and the relation F1 score (Eberts and Ulges, 2019), is used. Models based on SpERT (Eberts and Ulges, 2019) and DeepStruct (Wang C. et al., 2023) are employed to compare the performance between a span-based classification model and a generative model. To study the effect of the language-specific pre-training, a multilingual BERT (Devlin et al., 2019) and a Thai-specific WangchanBERTa (Lowphansirikul et al., 2021) are used in the span-based model. Lastly, in the generative settings, the pre-training model with mT5 (Xue et al., 2021) is compared to pre-training with our Thai UD dataset. All models are fine-tuned with the annotated event training set for the event extraction task.

Table 2 shows the micro-average F1 score for entity and relation extraction. For the span-based model, using language-specific pre-training substantially outperforms the multilingual one for both entity (66.97 vs. 44.58) and relation extraction (59.20 vs. 33.86). In our settings, generative models yield better results than the span-based ones. Notably, for entity extraction, the generative model trained with the multilingual pre-training can still outperform the language-specific span-based model (68.87 vs. 66.97). Finally, the best result in both entity and relation extraction is achieved when using language-specific UD pre-training (71.16 for entity extraction and 60.43 for relation extraction).

**Table 2:** The result of entity and relation extraction for event extraction of each model.

	**Model**	**P (%)**	**R (%)**	**F1 (%)**
Entity	SpERT (multilingual BERT)	37.05	55.93	44.58
	SpERT (Wangchanberta)	68.27	65.71	66.97
	DeepStruct (mT5)	78.22	61.52	68.87
	DeepStruct (UD)	78.96	64.76	71.16
Relation	SpERT (multilingual BERT)	25.44	50.58	33.86
	SpERT (Wangchanberta)	60.21	58.22	59.20
	DeepStruct (mT5)	50.69	65.49	57.15
	DeepStruct (UD)	53.56	69.33	60.43

## 5 Discussion

Compared to the baseline UD tagging, event annotation following our guideline is substantially faster. The decrease can be attributed not only to the fewer number of relations but also to the less complex annotation scheme that the annotators need to process. Annotating using our proposed guideline mostly follows the semantic structure of the sentence, eliminating the need to recognize minor syntactic relations like “case,” or “disclose.” The more complicated relations between clauses like “acl,” “advcl,” “csubj,” or “xcomp” are also omitted. In addition, event annotation treats multiple-word phrases as single units, eliminating the need to understand the intraterm connection. As a result, when developing the data for structural information extraction models, starting from semantic relations similar to the proposed event extraction could be more practical and time-efficient, especially for languages with no pre-existing structural training data.

From the subsequent span-based classification result, the model using language-specific pretraining outperforms the multilingual one in both entity and relation extraction, likely attributed to both language-specific and task-specific fine-tuning. Previous work has reported that using multilingual BERT performs substantially worse for low-resource languages, like Thai, as it does not benefit from cross-lingual transfer (Wu and Dredze, 2020) and shows that monolingual BERT-based models perform even worse for NER, POS, DEP tagging. In our case, we show that fine-tuning using task- and language-specific data offers an option to improve upon the monolingual BERT-based models.

When comparing the models in different settings, although the generative model with multilingual pretraining outperforms most of the span-based ones, it still lags behind the monolingual SpERT on the relation extraction task. This discrepancy is likely because the entity recognition task can benefit from the encoder-decoder architecture used in this work. A similar observation has also been previously reported (Wu et al., 2023). Nevertheless, specific downstream tasks must be taken into account when selecting candidate baseline models, as other types, such as masked LMs, could be computationally cheaper for domain-specific training.

In contrast to entity extraction, the relation extraction task could benefit more from the span-based two-step classification architecture. While SpERT inherently approaches relation extraction as a direct classification task, the generative-based method necessitates the simultaneous learning of relation generation with the identification of the entities of interest.

Lastly, when UD is included during the pre- training stage, the generative model outperforms in both tasks. Using UD information allows the model to learn the syntactic structure of the language, potentially aiding in the semantic inference of the subsequent relation extraction.

This result motivates the use of UD in conjunction with a more simplified event annotation framework when developing models for structure extraction, especially for low-resource languages. Although UD annotation is substantially more time-consuming, our work shows that including such information is likely beneficial to the subsequent semantic-related tasks.

## 6 Applications of event graphs

After obtaining the list of event attributes from the event extraction model, these sets of structured event information can be adopted to enhance other downstream tasks. In this section, we demonstrate the application of the extracted event graph to improve node classification in the retail banking product domain. Additionally, we explore the potential of transforming our event graph into a more generic knowledge graph where the types of relations are not constrained to only those present in our event annotation guideline.

The event graph in this experiment was constructed from the list of event triplets extracted using the UD-pretrained model from a set of 6,024 internal documents written in Thai, describing the details of financial products and services. This results in 69,801 nodes and 168,964 relations. Out of the total entity nodes, 500 nodes were selected and labeled into one of the 15 categories: “Process,” “Debit,” “Credit,” “Loan,” “Service,” “Promotion,” “System,” “Right,” “Fee,” “Insurance,” “Document,” “Contact,” “Account,” “Statement,” and “RewardPoint.” These nodes were selected such that the resulting 500-node sub-graphs were sufficiently connected (no disconnected graphs), and the numbers of each label were balanced. The averaged F1-score of 5-fold cross-validation of these 500-node sub-graph was then used to assess the performance of the model.

In the baseline model, only the text embedding derived from a pre-trained Thai language model, Wangchanberta (Lowphansirikul et al., 2021), was used. For our model, the node embedding derived from the event graph using Hash-GNN (Tan et al., 2020) was concatenated with the original text embedding as an additional feature.

Table 3 shows the averaged F1 scores of the model using text embedding or text+node embedding as features. The result shows an ~2 percentage point improvement (77.71% from 75.87%) when the model uses node embedding in conjunction with text embedding. This improvement underscores the significance of the relational information provided by our event graph using the simple Hash-GNN. To achieve further improvement, one could employ more advanced (though computationally more expensive) node embedding techniques, namely, GCN (Kipf and Welling, 2017) or GAN (Veličković et al., 2018). In addition to the improved performance, our node classification approach adaptable to other domains and can assist organizations in processing large textual data. A similar technique could be employed to categorize entity names present in internal documents, by labeling small subset samples and then using a classification model with the extracted event graph to incorporate contextual information.

**Table 3:** The comparison between models with and without node embedding as a feature.

**Model**	**F1-macro (%)**
W/o node embedding	75.87
W/ node embedding	77.71

Moreover, our extracted event graph can also be merged and reformatted to construct a more generic knowledge graph. Briefly, the procedure involves finding a pair of triplets such that the head entity of one pair is the same as the tail entity of the other pair. For example, the sentence “A criminal, previously exorenated, stole a car” would be converted into {subj, rel, obj} = {A criminal, stole, a car}. By merging the triplets afterward, the model is allowed to be trained under the constraint of recognizing only seven predefined relation types, yet allowing the extracted triplets to be rearranged to cover more generalized relations. Such a generalized knowledge graph can then be applied to assist in other domain-specific or language-specific information retrieval tasks, such as question answering on knowledge graphs (KGQA; Khongcharoen et al., 2022), or KG-enhanced LLMs (Pan et al., 2023).

## 7 Conclusion

In this paper, we introduced a streamlined event annotation framework that allows for substantially faster labeling over the baseline UD tagging. We propose that initiating the development of data for structural information extraction models with simple semantic relations, akin to event extraction, proves more practical, particularly for languages with no pre-existing structural training data.

Language-specific pretraining helps achieve better performance over the multilingual counterparts in both entity and relation extraction tasks. Notably, we underscored the importance of fine-tuning using task- and language-specific data to improve upon monolingual BERT-based models.

Under different settings, while the generative model with multilingual pretraining generally performs well, the span-based two-step classification architecture of SpERT shows a particular advantage for relation extraction tasks. The integration of UD information during the pre-training stage further improved the performance in both tasks, indicating a potential synergistic relationship between syntactic structure understanding and subsequent semantic inference.

Moreover, we leveraged the structured event information obtained from the event extraction model to improve node classification in the retail banking product domain. We also proposed a simple method for converting our event graph into a more generic knowledge graph that expands beyond our event relation types.

In conclusion, our research underscores the value of semantic-based event extraction, language-specific pretraining, and the integration of syntactic structure understanding through UD for improved performance in structural information extraction tasks. The methods we propose are not only efficient but also versatile, with potential applications in other domains, especially for developing similar structural training data for low-resource languages.

## Data availability statement

The data supporting the conclusions of this article will be made available by the authors, upon reasonable request.

## Author contributions

CS: Formal analysis, Investigation, Methodology, Validation, Writing – original draft. AT: Data curation, Formal analysis, Writing – original draft. TA: Data curation, Investigation, Writing – original draft. TC: Supervision, Writing – original draft, Writing – review & editing. ST: Conceptualization, Formal analysis, Supervision, Writing – original draft, Writing – review & editing. PB: Investigation, Supervision, Validation, Writing – original draft, Writing – review & editing.
