# Supplementary material for: Streamlining event extraction with a simplified annotation framework
Source: Front Artif Intell. 2024 Apr 29;7:1361483. doi: 10.3389/frai.2024.1361483 (PMC11089176; doi:10.3389/frai.2024.1361483)

frontiers

Supplementary Material

1 ANNOTATION GUIDELINE

The following section describe the annotation guideline used in this work. Note that, although the examples given here are written in English, the guideline was designed for Thai. The English examples were selected to exhibit similar syntactical structure. The guideline consists of the details of tagging an event and the design decision of the type. The annotation is split into two parts. First, entity annotation, which labels the the spans in the text along with its type. Second, relation annotation, which links the entities to their corresponding arguments.

1.1 Entities annotation

Entities are the graph node of an extracted event, which is the trigger and its arguments. In the Universal dependencies, the base unit that represents the graph node is a word. However, the entity or the node graph in this event extraction is the span of the text which can be a word or a phrase. With this definition, the annotator can ignore the syntactic relations among a clause like ‘det’, ‘case’, ‘acl’, ‘adcvl’, ‘subj’, ‘ccomp’, and ‘xcomp’ and decrease annotation time. Each entity’s length is determined based on its type, which will be described below.

The entities are categorized into seven types. *Action* is similar to the trigger of an event. Meanwhile, others are indicated as the arguments of an event. The types of arguments are designed to be similar to the types of Named Entities Recognition (**?**) to include a semantic meaning of an entity. Each type of entity is elaborated in the following subsection. The example of fully annotated sentence is shown below.

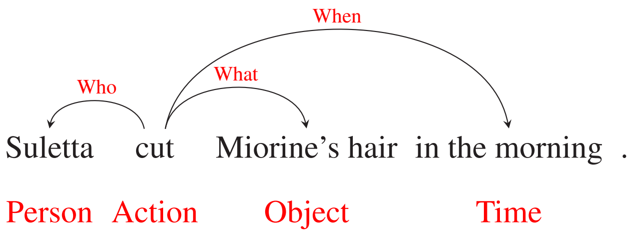

Action

Action is the main verb or the trigger of an event. This verb can be any type of verb which is not only an action verb, but also stative verb, process verb, action verb, experiential verb, and causative verb. However, the verb needs to be the core of an event. This verb will be considered as the predicate of other arguments that are related to the event.

Moreover, this type of entity can be serial verbs that have no conjunction between them. By tagging this serial verb as one span, the annotator does not need to consider syntactic relations among them, which includes many types of relations in UD like ‘compound’. As a result, the annotation time is descended.

Example:

- A gun belongs to the murderer. (Stative verb)
- A gun belongs to the murderer. (Process verb)

Object

Object is a noun phrase that is an argument of the verb that is *Action*. The noun phrase can be a subject, object, or complement of the verb. However, the noun phrase must be inanimate, including the object (both concrete and abstract) or a part and an organ of a living thing.

Example:

- Mr. John opens a door.
- A shirt shrink.
- Suletta cut Miorine's hair.
- A criminal drove a blue car to escape before dawn.

Person

Similar to *Object*, *Person* is a noun phrase that is an argument of the verb that is *Action*. The noun phrase can be a subject, object, or complement of the verb. In contrast to *Object*, this entity must be animate, including human and animal.

Example:

- Mr. John opens a door.
- Suletta cut Miorine's hair.
- A criminal drove a blue car to escape before dawn.

Organization

Organization is a noun phrase that is an argument of the verb (*Action*) including its subject, object, or complement. Different from *Object* and *Person*, this entity must be a group of persons or institutes that is established for a purpose or responsibility. In this case, this entity is usually a proper noun or named entity.

Example:

- A foundation gave survival bags to help a B community that was fired.
- D Group use 14,000 million baht to establish DF.

Location

This type of entity (*Location*) is the noun phrase of the core verb (*Action*). The entity is devised to indicate an area or a position that is related to *Action* or an event. In the other word, it can be both the start location of an event or the ending location of an event.

Example:

- A truck fell from a Rama IX bridge.
- A victim is imprisoned in a dark room for three days.
- William go gambling at Poi Pet last month.

Time

Time is the argument of the verb (*Action*) which is created to identify the time or order of an event. This type of entity can be a noun phrase, apposition phrase, or adjective clause. In this case, the whole clause or phrase is annotated as one span to decrease the time that is required to tag the syntactic relations among the word contained in the phrase like 'nummod', 'case', and 'det'.

Example:

- A criminal went robbing the bank this morning. (Noun phrase)
- The Thai president goes to Japan today. (Noun phrase)
- The victim is dead before 6 pm. (Adposition phrase)
- At 9 pm a warehouse is fired. (Adposition phrase)
- Before going to bed, you should brush your teeth. (Adjective clause)

Quantity

Sometimes, an event can contain an important number that is a necessary component to explain an event completely such as the amount of money that the victim is cheated. Thus, *Quantity* is created to determine a number or an amount that is related to the verb (*Action*). This type of entity is devised to indicate the value, money, statistics, or generic number. This unit is usually connected with the number by relation ’nummod’ in the UD. Moreover, this entity includes an integer, a ratio, a percentage, or a number in the written format. During the tagging, this entity includes the unit of the number.

Example:

- Each employee will get a special allowance 4,000 baht per month
- A new customer will get 66% discount.
- A customer withdraws fifteen thousand baht.

1.2 Relation annotation

A relation is a link between two nodes or entities in the graph. To show the semantic meaning among the entities, relations are designed to answer mainly WH questions: what, who, when, and where. The relation connects the verb or *Action* of an event to its argument similar to Universal dependencies that starts with the main verb or the root of the graph. In the event extraction, only semantic relations are needed to be considered, some syntactic relations like ‘det’, ‘case’ or relations linking between clauses including ‘acl’, ‘adcvl’, ‘subj’, ‘ccomp’, and ‘xcomp’ can be ignored. Therefore, the annotator can tag an event with less effort compared to tagging an UD graph. Relations are categorized into seven types, which are explained as follows.

What

What is a relation that links between an action *Action* of an event to an entity type *Object* as its argument. In this case, *Object* can be a subject or a direct object of a sentence or an instrument in the event depending on *Action*.

Example:

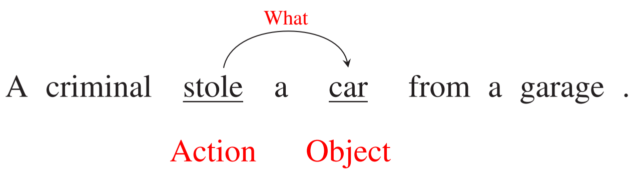

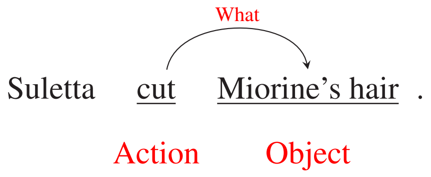

Who *Who* is a relation that links between an action *Action* of an event to an entity type *Person* or *Organization* as its core argument. In the meaning *Person* or *Organization* is a subject or a direct object from an *Action* in the event to capture only a necessary part of an event. Thus, some indirect objects that are not mandatory to explain an event can be ignored, so the annotator can cut off that part and decrease an annotation effort.

Example:

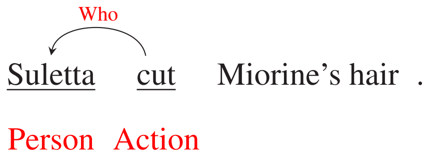

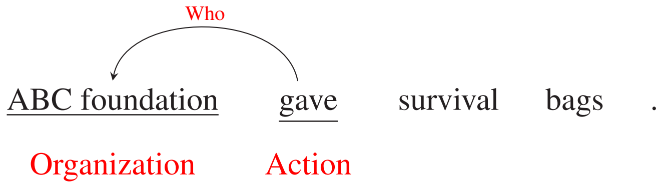

When

When is a relation that links between an action *Action* of an event to an entity type *Time* as its argument. Considering in a meaning, this entity *Time* is determined as a non-core argument of an *Action* in the event. This entity (*Time*) can be both a small event that indicates the time or an adjective phrase of an *Action*.

Example:

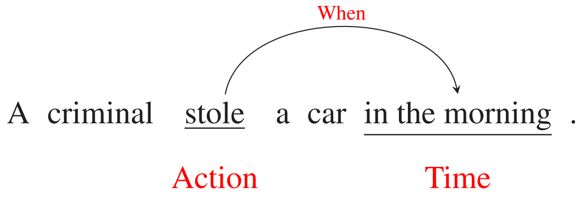

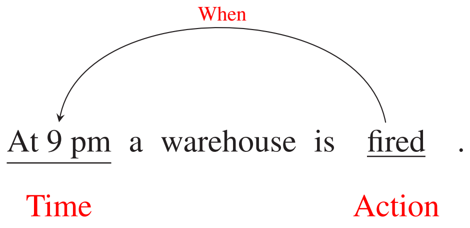

Where

Where is a relation that links between an action *Action* of an event to an entity type *Location* or *Organization* as its argument. Considering the meaning, this entity indicates the location of an event. In other words, this entity is a non-core argument where the event happened.

However, *Object* is sometimes indicated as a location of an event. To handle this case, this relation also connects the entity *Object* to its *Action*. This entity *Object* will convey the meaning as a location or a place, such as an ATM or an application. In other words, the entity *Object* will be a non-core argument of the *Action* that means a location or a place only.

Example:

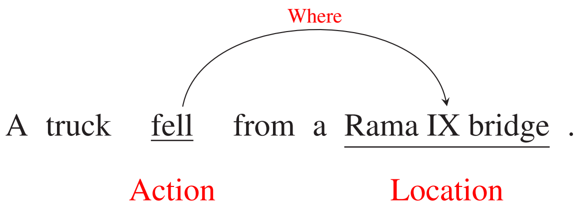

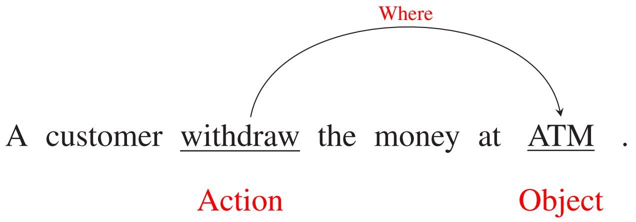

Same-unit

In the sentence, the main verb or *Action* is a serial verb, phrasal verb or its compounds, but they are sometimes not located next to each other, such as ”put them up”, which can have its object like ”them” between the word ”put” and the word ”up”. Thus, the relation that indicates these verbs and components are connected to each other indicates that they are needed to connect to each other to convey the complete meaning.

Example:

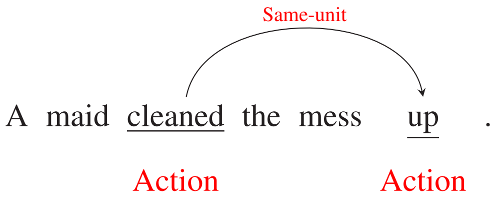

Benefit Some events show that only capturing a direct object using a relation ”Who” cannot convey a complete message to explain an event. Especially, when the indirect object gets a benefit from an event. Thus, *Benefit* is created for representing this information. *Benefit* links between an action *Action* of an event to an entity type *Person* or *Organization* as its argument that gets the benefit from an event. This relation is different from a relation *Who* in that its entity type is an indirect object that acquires the benefit from an event only.

Example:

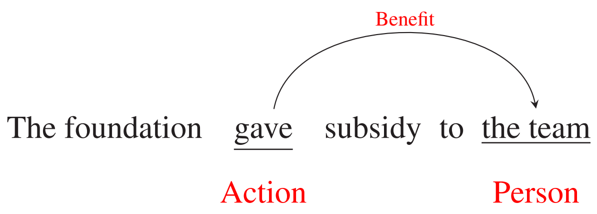

Value *Value* is a relation that is created to connect between the entity type *Quantity* and the action of an event to indicate the important number that is required to explain the event. In other words, this entity *Quantity* will be a non-core argument of the action that determines the value related to the action.

Example:

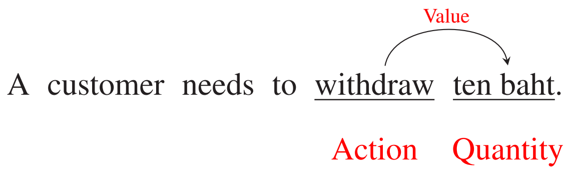

Supplement: Supplementary file 1 [file Data_Sheet_1.PDF]
